# Investigating the impact of endemic mosquitoes and invasive *Aedes* species on the circulation of *Dirofilaria* nematodes

**DOI:** 10.1186/s13071-025-06901-0

**Published:** 2025-07-07

**Authors:** Zsaklin Varga, Rebeka Csiba, Ágota Ábrahám, Dorina Pásztor, Csongor Németh, Gábor Kemenesi, Krisztián Bányai, Kornélia Kurucz

**Affiliations:** 1 Szentágothai Research Centre, National Laboratory of Virology, University of Pécs, Pécs, Hungary; 2 Institute of Biology, Faculty of Sciences, University of Pécs, Pécs, Hungary; 3 Department of Dermatology, Venereology, and Oncodermatology, Medical School, University of Pécs, Pécs, Hungary; 4 Department of Pharmacology and Toxicology, University of Veterinary Medicine, Budapest, Hungary; 5 Department of Medical Biology, Medical School, University of Pécs, Pécs, Hungary; 6 National Laboratory for Health Security, University of Pécs, Pécs, Hungary

**Keywords:** Human infection, Filarioidea, Vector-borne pathogens, Climate change, Tiger mosquito, Zoonotic disease, Blood-meal analysis, *Culex pipiens* biotype

## Abstract

**Background:**

Mosquito-borne pathogens represent a growing challenge driven by environmental changes and the spread of invasive *Aedes* mosquitoes. Among pathogens endemic in Europe, *Dirofilaria* nematodes are of particular concern due to the increasing number of human infections. To understand their zoonotic potential, we aimed to assess the prevalence of filarioid nematodes in mosquitoes and reveal the potential vector species, considering their feeding behavior.

**Methods:**

Mosquitoes were collected from urban/suburban environments (Pécs, Hungary) in 2022–2023 and tested for the presence of filarioid nematodes using polymerase chain reaction (PCR) and DNA sequencing. Estimated infection rates with 95% CI were calculated, and descriptive statistics were applied.

**Results:**

Among 1015 tested mosquito pools (belonging to 21 species), 30 were positive for filarioid nematodes, including *D. repens*, *D. immitis*, *Setaria tundra*, and *Setaria labiatopapillosa*, with the highest prevalence and widest distribution of *S. tundra*. We revealed hotspots in the city where multiple filarioid species occurred. The presence of *D. repens*, relevant for humans, was confirmed in urban and suburban areas and near human infection cases. Among mosquitoes, *Aedes vexans* showed the highest positivity, harboring all identified parasites, while invasive *Aedes albopictus* and *Aedes koreicus* showed minimal/no infections. Moreover, we identified *S. labiatopapillosa* in urban areas in Hungary for the first time.

**Conclusions:**

Our findings highlight the potential role of *Ae. vexans* in transmission dynamics, while no evidence was found for the contribution of invasive mosquitoes, likely due to local environmental and behavioral factors. Our results emphasize the need for targeted vector monitoring and research to understand mosquito-borne parasites’ epidemiology and public health implications, particularly in regions affected by invasive mosquitoes.

**Graphical Abstract:**

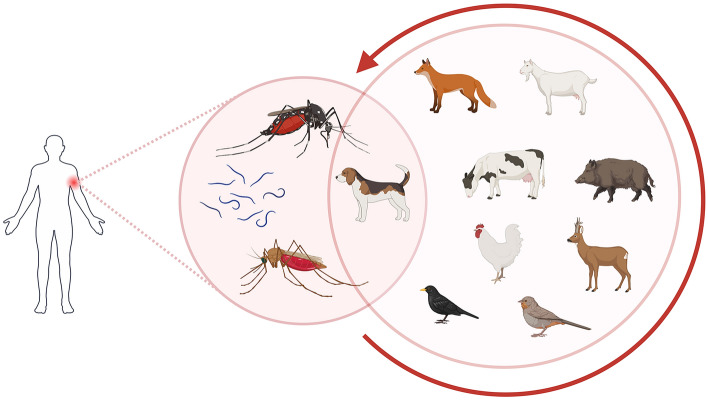

**Supplementary Information:**

The online version contains supplementary material available at 10.1186/s13071-025-06901-0.

## Background

The introduction of exotic species to new territories has a profound impact on native species and biodiversity. Moreover, in the case of invasive species, their economic implications are also significant. They impact tourism or even public health systems, necessitating increased efforts for control and management strategies. From this point of view, invasive mosquitoes arriving from Asia pose one of the most significant problems in Europe. Notably, the species *Aedes albopictus* (Skuse, 1894), *Aedes japonicus japonicus* (Theobald, 1901), and *Aedes koreicus* (Edwards, 1917) show a wide distribution on the continent with the potential of transmitting a variety of pathogens [1–3]. As a result of their invasion, they play a significant role in the emergence of vector-borne diseases and can also influence the dynamics of endemic pathogens [4, 5]. It means substantial public health concerns, since those mosquito species not only can transmit exotic pathogens such as dengue, chikungunya, Zika, and yellow fever virus, but are also competent vectors of West Nile virus or onchocercid nematodes such as *Dirofilaria immitis* and *Dirofilaria repens*, endemic in Europe [3–6].

Among the mentioned species, *Ae. albopictus* was first detected in Europe in 1979 (in Albania), and since then, it has been recorded in almost all countries [7]. Human activities, such as trade and transport, have facilitated its spread, allowing it to establish populations across the whole continent. Similarly, *Ae. j. japonicus* has progressively spread across Europe since its initial detection in the early 2000s [8]. The expansion of *Ae. koreicus* is also driven by similar human-mediated factors. However, it shows a less intensive spreading and is predominantly established in specific temperate areas, indicating a more gradual and regionally limited dispersal pattern [9]. Hungary is one of the few countries where all three species are established: *Ae. j. japonicus* emerged in 2012 and *Ae. albopictus* was described in 2014, while *Ae. koreicus* in 2016 at the earliest [7, 10, 11]. The country’s unique geographic location in the Carpathian Basin, where temperate and Mediterranean climatic influences converge, provides favorable conditions for spreading invasive mosquitoes and the potential emergence of pathogens they can transmit. The increasing presence of these species makes Hungary an ideal region to investigate their role in circulating vector-borne pathogens, thereby serving as an essential model for Central Europe.

In this region, *Dirofilaria* parasites are the most representative mosquito-borne pathogens, causing both animal and human infections. They primarily affect Canidae, while humans are considered incidental hosts. However, the increasing number of reported human dirofilariasis cases in the last decades reflects the growing significance of an emerging pathogen with zoonotic potential [12, 13]. In Hungary, human subcutaneous and pulmonary dirofilariasis cases are reported each year [14–17]. However, the number of infections is probably higher than reported, as the parasite does not always cause severe symptoms. Notable, in the city of Pécs (a regional center in the southwestern part of Hungary), several human cases of *D. repens* infection have been reported since 2017 [14, 16], which coincides with the successful establishment of *Ae. koreicus* in the city (since 2016), a potential vector for *Dirofilaria* species [6, 11]. Although monitoring invasive mosquito species has significantly improved in the last few years, their vector potential in the region remains unknown. In addition, the surveillance of filarioid parasites in vector organisms, i.e., mosquitoes, is also less investigated in Hungary. To enhance knowledge of the factors contributing to human infections, we aimed to assess the prevalence of filarioid nematodes in local mosquito populations and identify which mosquito species may facilitate the circulation of these parasites in urban environments, with particular emphasis on invasive *Aedes* species. As mosquitoes acquire pathogens primarily by feeding on infected reservoir hosts, investigating their host feeding patterns helps identify critical points of parasite transmission. Indeed, mosquitoes’ feeding patterns vary between species and can also vary between geographically distinct populations of the same species, indicating that regional or population-level adaptations may significantly affect host utilization [18]. Therefore, we also intended to conduct blood-meal analysis from field-collected mosquitoes to reveal which host species they preferentially feed on. This approach may help to understand vector–host interactions and transmission dynamics and evaluate the potential risks of human *Dirofilaria* infections in the given area.

## Methods

### Mosquito collection and sample preparation

**Figure 1:**
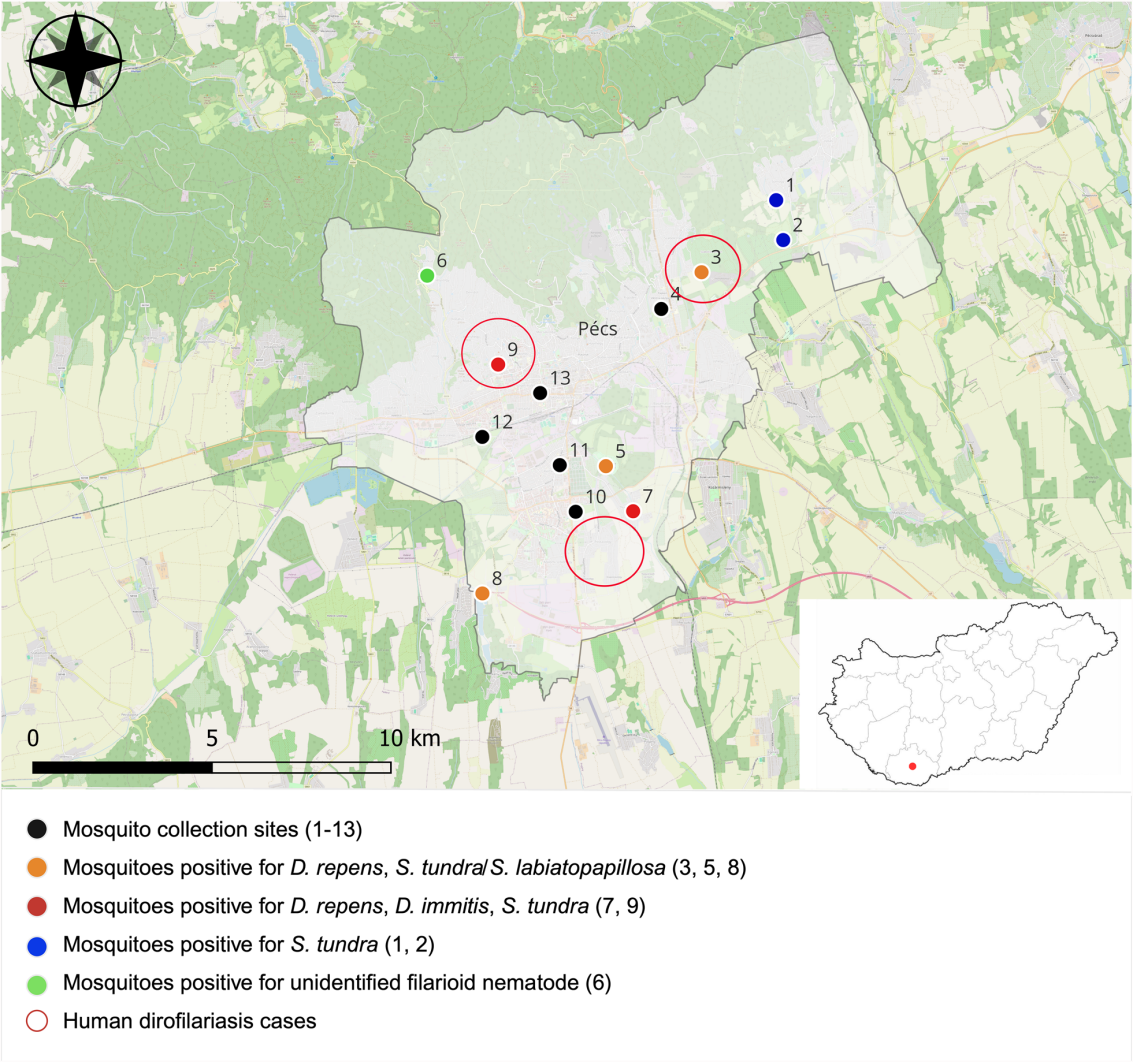
Mosquito sampling sites (1–13), distribution of detected mosquito-borne parasites (*Dirofilaria immitis*, *Dirofilaria repens*, *Setaria tundra*, and *Setaria labiatopapillosa*), and human dirofilariasis cases in the city of Pécs (Baranya county, Hungary). The map was created using the QGIS free software version 3.34.13 (https://www.qgis.org)

Mosquito samples were collected within the framework of a local monitoring program in the city of Pécs (and its outskirts), Hungary (46°06′27.31″ N, 18°12′24.17″ E), between May 2022 and October 2023. Sampling sites (altogether 13 sites) covered mainly urban and suburban areas, including private gardens, urban green areas with apartment blocks around, a botanical garden, an animal shelter, and outdoor recreational parks with forestry areas around, where mosquito–human interactions are common (Fig. 1). Trapping of mosquitoes was carried out with Heavy-Duty Encephalitis Vector Survey (EVS) traps (Bioquip, Rancho Dominguez, CA, USA) operating with dry ice as CO_2_ attractant once per week, overnight. The trap nets, including the collected live mosquitoes, were transported to the laboratory by car (immediately after collection), and euthanized by freezing at −20 °C. Female mosquitoes were then selected and morphologically identified one by one at the species level [19, 20]. At the same time, blood-fed mosquitoes (all the mosquito individuals containing blood meals in their abdomen) were determined and selected from all non-fed specimens. Then, following standard practice in field studies screening for pathogens, female mosquito individuals were pooled per collection date, site, and species, with a maximum of 10 individuals per pool (min. 1–max. 10). A different approach was applied in the case of invasive *Aedes* mosquito species, which were processed individually (not pooled) because they were also used for genetic analysis as part of the monitoring program. Similarly, blood-fed specimens were also processed on an individual basis since we were interested in the identification of blood source at an individual level. For this, the abdomens were separated from the head–thorax part using sterile tips, then used separately for further analyses (see Screening of filarioid nematodes and Blood-meal analysis sections).

For the current study, we involved only a subset of all collected mosquito samples: a total of 1015 pools were selected systematically in terms of representing all collection sites, months, and species (*Aedes*, *Culex*, *Culiseta*, *Anopheles*, *Coquillettidia* genera) captured during the monitoring program, including invasive *Aedes* species and blood-fed mosquitoes as well.

In the case of *Culex pipiens* s.s., the genetic forms (*Cx. pipiens* biotype *pipiens*, *Cx. pipiens* biotype *molestus* and their hybrids) were retrospectively determined using the rapid assay by Bahnck and Fonseca [21], applied on pools containing a single specimen only. For detailed information on investigated mosquito pools, see Additional file 1: Dataset S1.

### Screening of filarioid nematodes

For filarioid screening, the samples (whole mosquito bodies or head–thorax part in the case of blood-fed individuals) were homogenized in 100 µL PBS (phosphate buffered saline) using two glass beads with a Qiagen TissueLyser II (Qiagen, Germany) machine. For nucleic acid extraction, the Zymo Quick-DNA Miniprep Plus Kit (Zymo Research, USA) was used according to the manufacturer’s instructions. The samples were screened for filarioid nematodes using the protocol previously described by Czajka et al. [22]. A TaqMan PCR (primer set targeting a 94-bp long fragment of the 12S rRNA gene) was performed with GoTaq G2 Flexi DNA polymerase kit (Promega, Wisconsin, USA), with a 25 µL final volume mix containing 2–2 µL primers (10 µM), 1 µL probe (10 µM), and 5 µL template. The thermal cycling program was performed on a MyGo Mini S Real-Time PCR machine (IT-IS Life Science Ltd.) with the following conditions: 95 °C for 5 min for denaturation and 50 cycles of 94 °C 30 s, 50 °C 20 s, and 72 °C 20 s for amplification. Then, to identify the species of the parasites, TaqMan PCR-positive samples were further examined by conventional end-point PCR (primer set amplifying a 670-bp region of mitochondrial COI) [23], using Gotaq G2 Flexi DNA polymerase kit (Promega, Wisconsin, USA), with 25 µL of final PCR mix contained 0.5–0.5 µL forward and reverse primers (100 µM) and 5 µL template. The thermal cycling program included 95 °C for 2 min of denaturation, followed by 40 cycles of 94 °C for 30 s, 52 °C for 30 s, and 72 °C for 50 s for amplification and 72 °C for 5 min for final extension. The PCR product was run on a 1.5% agarose gel, and then DNA was purified from the gel using Monarch PCR & DNA Cleanup Kit (New England Biolabs, Massachusetts, USA). The purified DNA was sequenced by Eurofins Genomics (Eurofins Genomics, Germany) and Microsynth (Microsynth AG, Switzerland) using Sanger sequencing. Then, the parasite species was identified on the basis of NCBI BLAST searches.

### Blood-meal analysis

Among the samples we involved in the current study, 63 specimens were suitable for blood-meal analysis: 46 specimens of native mosquito species (*Aedes vexans*, *Cx. pipiens* s.s., *Culiseta annulata*, *Ochlerotatus annulipes*, *Och. caspius*, *Och. sticticus*, *Och. cantans*, *Anopheles maculipennis*) and 17 specimens of the invasive *Ae. koreicus* that contained blood in their abdomens that were dissected before (see Sects. Mosquito collection and sample preparation). For the molecular identification of blood meal origin, i.e., host species (PCR followed by sequencing and NCBI BLAST search), previously described protocols were applied, using the QIAamp DNA Micro kit (Qiagen, Germany) and the Gotaq G2 Flexi DNA polymerase kit (Promega, Wisconsin, USA) [24, 25].

### Data analysis

To estimate the prevalence of filarioid nematodes in mosquitoes, estimated infection rates (EIR) with 95% confidence intervals (CI) were calculated by months using the Epitools software (available from AusVet Animal Health Services, https://epitools.ausvet.com.au/), considering differences in pool size (number of mosquito individuals per sample) and assuming 100% sensitivity and specificity [26]. Due to the relatively small number of filarioid-positive mosquito pools, the effect of climatic factors (mean temperature, precipitation) or mosquito seasonality on infection could not be assessed. Therefore, descriptive statistical analysis was applied.

## Results and discussion

Among the tested 1015 mosquito pools, a total of 30 pools were positive for filarioid nematodes (EIR 0.007; 95% CI 0.005–0.009). In 6 pools, *D. repens*; in 2 pools, *D. immitis*; in 18 pools, *Setaria tundra*; and in 1 pool, *Setaria labiatopapillosa* (Filarioidea: Onchocercidae) were identified at 8 sampling sites (Table 1, Fig. 1). The latter species, *S. labiatopapillosa*, was detected for the first time in Hungary. In three cases of positive pools, neither the parasite species nor the genus could be identified.

**Table 1:** Summary of investigated mosquito species with the number of tested (negative and positive) pools, including parasite species identified in positive samples

Mosquito species	No. of tested mosquito pools		Parasite species
	Negative	Positive	
*Aedes albopictus*	48	0	
*Aedes cinereus*	2	0	
*Aedes hungaricus*	1	0	
*Aedes koreicus*	316	1	1 *Setaria tundra*
*Aedes koreicus/japonicus*	6	0	
*Aedes rossicus*	2	0	
*Aedes vexans*	269	11	1 *Dirofilaria immitis*
			4 *Dirofilaria repens*
			1 *Setaria labiatopapillosa*
			5 *Setaria tundra*
*Anopheles claviger*	13	1	1* Setaria tundra*
*Anopheles hyrcanus*	1	0	
*Anopheles maculipennis*	7	2	2* Setaria tundra*
*Anopheles plumbeus*	9	0	
*Coquillettidia richiardii*	22	2	1 *Filaria spp.*
			1 *Setaria tundra*
*Culex martinii*	2	0	
*Culex pipiens*	118	6	1 *Dirofilaria immitis*
			1 *Dirofilaria repens*
			1 *Filaria spp.*
			3 *Setaria tundra*
*Culex theileri*	2	0	
*Culiseta annulata*	38	1	1* Dirofilaria repens*
*Ochlerotatus annulipes*	18	1	1 *Setaria tundra*
*Ochlerotatus cantans*	11	2	2 *Setaria tundra*
*Ochlerotatus caspius*	6	0	
*Ochlerotatus caspius/dorsalis*	1	0	
*Ochlerotatus geniculatus*	6	0	
*Ochlerotatus rusticus*	39	2	1* Filaria spp.*
			1 *Setaria tundra*
*Ochlerotatus sp.*	2	0	
*Ochlerotatus sticticus*	46	1	1 *Setaria tundra*

Several of the mosquito species we investigated in the current study are known to be able to transmit *Dirofilaria* nematodes, particularly *Ae. vexans*, *Cx. pipiens* s.s., and the invasive *Ae. albopictus*, *Ae. koreicus*, which are species that generally prefer human proximity and feed on both animals and humans [5, 6, 27–30], and thus may serve as bridge vectors for pathogens.

**Figure 2:**
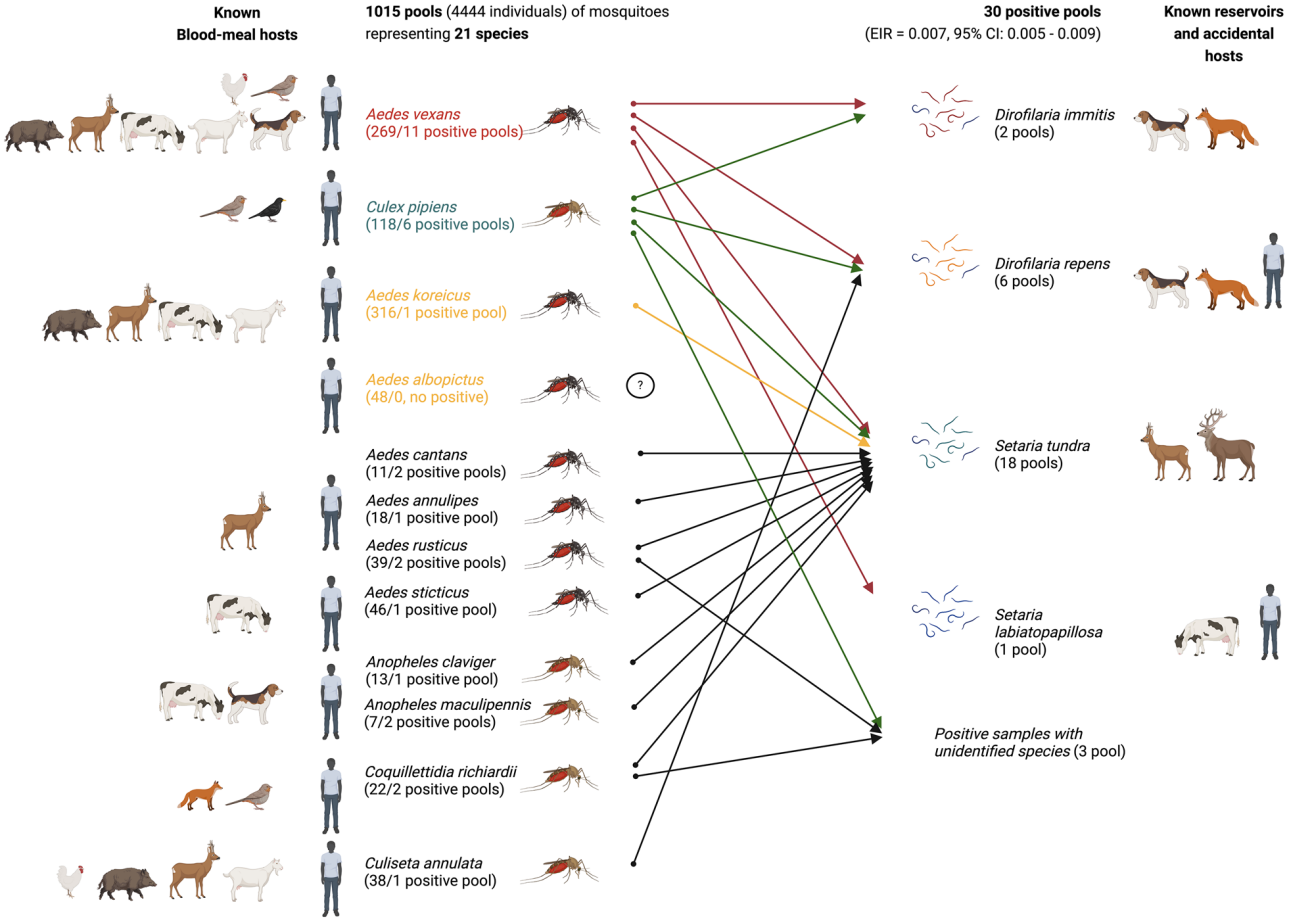
Overview of mosquito species investigated in this study, detected parasites, their potential reservoirs and accidental host organisms known from the literature, and the known feeding patterns of the given mosquito species based on our current results and literature cited in the manuscript. Created in BioRender. Kurucz, K. (2025) https://BioRender.com/xidachb

In our study, most of the positive pools (in 11 cases) contained *Ae. vexans*, an endemic mosquito native to Hungary, as well as widespread and abundant species in the country [31]. It also represented high densities near human infections in Pécs on the basis of our faunistic monitoring measures. We found all the identified parasites mentioned above (*D. repens, D. immitis, S. tundra, S. labiatopapillosa*) in this mosquito species (Table 1, Fig. 2), which suggests *Ae. vexans* can harbor a variety of parasites in the investigated area. Regarding its feeding preferences, *Ae. vexans* are known to exhibit opportunistic behavior, with host selection influenced by local availability. They preferentially feed on mammals, including domestic livestock, wild animals, and humans, while occasionally, they may also feed on birds [24, 25]. In our investigation, among the 28 *Ae. vexans* specimens, we could analyze for the blood-meal host: 32% (9 individuals) fed on humans; 25% (7 individuals) fed on the wild game, including wild boar (*Sus scrofa*), roe deer (*Capreolus capreolus*), and other cervids (*Cervidae,* not determined at species level); and 14% (4 individuals) fed on domesticated animals as cattle (*Bos taurus*) and goat (*Capra hircus*); while in 1–1 cases we identified domesticated cat (*Felis catus*) and a bird species, black redstart (*Phoenicurus ochruros*) as the origin of the blood-meal. Unfortunately, in six cases, we could not identify the host (marked as “no result”). Remarkably, a single specimen that fed on a goat (based on the blood-meal analysis from the abdomen) was positive for *S. tundra* (based on filarioid screening from the head–thorax part of the mosquito) (Additional file 1: Dataset S1). Our findings align with the anthropophilic and mammalophilic feeding tendencies while reflecting the host diversity representative of the investigated area. Considering the feeding habits, rapid reproduction, and flying capabilities of *Ae. vexans*, it may play a significant role in the circulation of *Dirofilaria* species and their transmission to humans in the region.

A similar diversity of parasites was observed in *Cx. pipiens* s.s. mosquitoes, with six pools testing positive for *D. immitis*, *D. repens*, and *S. tundra* as well (Table 1, Fig. 2). However, the vector potential of this mosquito species may depend on the feeding patterns of different biotypes, which vary in their ecology and behavior, although they can form hybrids [32, 33]. The common assumption is that the biotype *pipiens* is thought to be highly ornithophilic and the biotype *molestus* highly mammalophilic, while hybrids of them show an indiscriminate biting behavior, but the host selection of mosquitoes is mostly directed by the availability of hosts in addition to innate preference [34]. In the city of Pécs, we found that all biotypes, including hybrids, occur in urban environments, although we only had the opportunity to analyze the host of one blood-meal, which was a bird (black redstart) (Additional file 1: Dataset S1). Indeed, this single result is not appropriate to draw any conclusion; in a region of high hybridization, both *pipiens* and *molestus* forms showed a high preference to avian blood meals, heightening the changes of being bridge vectors [35].

Regarding *Ae. koreicus,* only one pool was positive for *S. tundra*, while no positive samples were found in *Ae. albopictus* (Table 1, Fig. 2). Both mosquito species are considered potential vectors for *Dirofilaria* species. The vectorial capacity of *Ae. koreicus* is assumed on the basis of laboratory experiments [6, 36], but in a previous study in the same area (in the city of Pécs), *D. repens* was detected in captured specimens of the species [37]. However, the evidence for transmission of the parasite by *Ae. albopictus* has been shown in the Italian population [5, 38, 39], coupled with the increasing prevalence of human dirofilariasis in Italy [13]. In the current study, the local populations of invasive mosquito species might have a low infection rate, possibly due to their feeding behavior, limited exposure to infected animal hosts, or even the environmental conditions, such as temperature or humidity, specific to the study area. Invasive mosquitoes are known to prefer urban environments and feed on humans; however, they are considered to be mammalophilic and can feed on different mammal hosts according to local abundance [40]. We obtained similar results, as we found that among the 17 *Ae. koreicus* specimens (involved in the blood-meal analysis), 59% (10 individuals) fed on humans and 29% (5 individuals) fed on wild game (wild boars and cervids, not determined at species level), while 12% (2 individuals) fed on domesticated animals (cattle and goat) (Additional file 1: Dataset S1). Due to the limited number of blood-fed specimens, we could not involve *Ae. albopictus* specimens in the current blood-meal analysis; however, on the basis of our preliminary results on 25 specimens originating from a different urban area (the city of Barcs, Somogy county, Hungary), they fed exclusively (100%) on humans (unpublished data). Notably, the diet diversity of *Ae. albopictus* depends on its invasion history, namely earlier-established populations exhibiting broader host diversity than those introduced more recently [41]. In general, mosquitoes that feed on a broader range of hosts might facilitate greater parasite dispersal, and if mosquitoes feeding on animal reservoirs also frequently bite humans, there is an increased risk of zoonotic spillover to humans. For an overview of revealed parasite–vector–host interactions, see Fig. 2.

Seasonal or temporal variations in mosquito activity or parasite transmission dynamics might also have influenced the infection rates, potentially favoring other mosquito species, i.e., *Ae. vexans* in the studied area and seasons. During the study period, we observed varying prevalence of filarioid infestations across the months, with the highest positivity recorded in June and July in both years (in May 2022: EIR 0.0189, 95% CI 0.0011–0.0804; June: EIR 0.0193, 95% CI 0.0112–0.0307; July: EIR 0.0344, 95% CI: 0.0108–0.0783; in August, September, and October no positive samples were found; in June 2023: EIR 0.0015, 95% CI 0.0002–0.0046; July: EIR 0.0107, 95% CI 0.0043–0.0216; August: EIR 0.0068, 95% CI 0.0011–0.0207; in May, September, and October no positive samples were found). This observed pattern is consistent with the known seasonality of filarioid in mosquitoes and the phenology of mosquitoes [37, 42]. However, due to the low number of positive samples, there is no confirmed correlation between the temporal dynamics of mosquito populations and the infection rate. Regarding spatial distribution, parasites were detected at 8 of the 13 sampling sites, showing different distributions by parasite species and highlighting some hotspots in the city with co-occurrence of multiple filarioid species at the same sampling sites. From this point of view, a private garden with livestock animals (site no. 7) and the botanical garden at a university campus (site no. 9) are notable, where both *D. immitis* and *D. repens* as well as *S. tundra* were found (Fig. 1). Also notably, the presence of *D. repens*, relevant for the detected human cases, was confirmed at five sites, either in urban and suburban areas (in the botanical garden, private gardens, and outdoor recreational parks with forestry areas around) or in proximity to areas with reported human cases (Fig. 1). This suggests potential implications for both human and animal dirofilariasis as well.

In addition, our study demonstrated the presence of *S. tundra* and *S. labiatopapillosa* in urban areas. These parasites are widely distributed across the Northern Hemisphere and have veterinary importance since they primarily infect wild ungulates, such as cervids and domestic ruminants [43]. Regarding human relevance, neither *Setaria* species is known as a significant human pathogen. However, a few cases of human filariasis due to *S. labiatopapillosa* were found in Bucharest, Romania [44]. In addition, considering the wide distribution of *S. tundra* in the studied area, and the proximity of human filariasis cases in the city, its zoonotic potential can not be excluded [22, 44, 45]. Furthermore, they are ecologically relevant for understanding the dynamics of mosquito-borne pathogens.

## Conclusions

The main focus of this study was to reveal the potential role of invasive mosquitoes, i.e., *Ae. koreicus* and *Ae. albopictus* established recently in the region, in the circulation of *Dirofilaria* parasites and their transmission to humans. Since these species showed minimal or no infection in the study, no conclusions can be drawn regarding their involvement in human dirofilariasis. Our work provides valuable insights into the local diversity of mosquito-borne filarioid parasites, highlighting key potential mosquito vectors, specifically *Ae. vexans* and *Cx. pipiens* s.s., as well as hotspots in the investigated area. Our findings align with known mosquito phenology and demonstrate the importance of habitats where human–animal interactions frequently occur in maintaining pathogen circulation. Detecting veterinary-relevant parasites, such as *S. tundra*, and particularly the first detection of *S. labiatopapillosa* in the Hungarian urban areas, further emphasizes the ecological relevance of these species in understanding mosquito-borne pathogens dynamics. However, their direct impact on human health remains minimal. Overall, our work highlights the importance of targeted surveillance and the need for integrated vector management strategies to mitigate the risks posed by filarioid parasites to both human and animal health.

## Supplementary Information

Additional file 1. Dataset S1. Data on field collection of mosquitoes involved in the present study, including the screening of filarioid nematodes, blood-meal analysis, and biotype (genetic form) determination of *Culex pipiens*.

## Data Availability

Data supporting the main conclusions of this study are included in the manuscript.
